# Translations

**Published:** 1882-03

**Authors:** Seth N. Jordan

**Affiliations:** Columbus, Ga.


### Translations.

---

BY SETH N. JORDAN, M.D., COLUMBUS, GA.

---

*From Foreign Journals for the Atlanta Medical Register.*

---

*Corsets for Scoliosis. v. Bergman. Hospital Reports, No.* 35, *p.* 345.—In the place of plaster of Paris von Bergman uses poroplastic felt for the construction of corsets in spinal curvature. This felt has the advantage of adapting itself to the body by warming. It is best to have a number made at the manufactory, in order to be able to fit the different figures. The jackets are adapted on the suspended body. Like Sayre, von Bergman leaves a wad of cotton on the concave side of the curvature, which, after the corset is hard, is to be removed, thus allowing ample expansion of the thorax, while on the other hand, the convex side of the curvature, by means of the bandage, is subjected to continuous pressure. The only fault of the felt corset is its price, costing about eight to ten dollars; however, this is equalized by the fact that they can be repeatedly used. As a general thing the jacket should be changed every two months. Corsets provided with a posterior lacing string are preferred as they allow a change at any time. Not until there is evident improvement should we begin with gymnastic-orthopedic exercises. The felt corset is of not the same value in cyphosis, which is due to the presence of spondylitis. Here absolute rest in bed is alone to be recommended.

---

*Pathological Conditions in Cases of Death by Hanging. Med. Jurisprudence, Vol. 35, p. 248. Hospital Reports. A. Lesser.* Lesser's observations extend over fifty cases. In three cases the only lesion was on the skin. The appearance left by the rope of a sunken furrow was still present, and the skin was anemic, while the adjoining surface was markedly hyperæmic. In a fourth case hemorrhages under the sterno-cleido-mastoid muscle were observable. Five cases concerned lesions of the deeper soft parts alone, viz: in one case extravasation in the platysma myoides; in two, the same on the perichondrium of the thyroid cartilage and the ligamentum hyo-thyroideum; in another, hemorrhage in close proximity to the thyroid; and finally several miliary hemorrhages in the mucous membrane of the pharynx.

Accompanying each of the lesions of the skin was a rupture of the spinal column with extravasation of blood between; once rupture of the thyroid cartilage, once fracture of the hyoid bone, with hemorrhage between the fractured ends and the periosteum. In one case, in addition to the skin lesion, there existed an hemorrhage in the adventitia of the carotids, also in the region of the thyroid cartilage, and besides this fracture of the hyoid bone and extravasation between its ends and the periosteum. Twelve cases were lesions of the soft parts about the hyoid bone and larynx; relating to hemorrhages: in a subcutaneous lymphatic gland, on the hyo-thyroid ligament, in the adventitia of the carotids, on the thyroid cartilage, in the hyo-glossus muscle, behind the hyoid bone in the mucous membrane of the pharynx, in the omohyoid muscle, in the retropharyngeal tissues; in five cases were fractured both superior ends of the hyoid bone; five times the right alone, and once the left alone. In six cases the hyoid bone was alone broken. In twenty-one cases there was scarcely any evidence that suspension had occurred to the body *intra vitam*. Neither the number nor the gravity of the injuries found stand in a direct ratio to the size of the rope or to the strength, which, to appearances, would naturally come into action by strangulation.

In many instances there was neither fracture nor hemhorrhage of or near the hyoid bone. An explanation of this can be found in the fact that the rope exercises an equable pressure on these parts often until all circulation ceases, giving this portion a very pale appearance.

---

*The Etiology of Scurvy. Amburger, Wochenschrift, Berlin,* 1881, *No.* 25.—Scurvy appears endemically in Petersburg. Most of the cases fall in the months of March and May, this being the time of the arrival of barks filled with badly nourished seamen. Errors in diet, Amburger considers the only source of scurvy. Want of fresh meats and vegetables, indigestible canned goods; all of these are so considered.

Scurvy also occurs often after fasting for several days. Moisture and influence of the weather are of no etiological importance. The author could neither convince himself of the miasmatic, infectious or contagious nature of scurvy.

---

*The Treatment of Fracture of the Ribs with Plaster of Paris Bandages. Englisch, Med. Presse, No.* 31, 1881.—This treatment, although generally, for some unaccountable reason, condemned, has been treated in thirty-eight instances by our author. The jacket is applied at an intermediate station between respiration and expiration, and it is of great importance that the circular turns be not too tightly drawn. The time of application varies from 2–8 days after the injury. In complicated cases there is no necessity for waiting at all. In every case the advantage of this bandage in allaying pain and admitting transport was conspicuous. Bloody sputum and inflammatory results were seldom observed. In cases of cutaneous emphysema, rapid disappearance of same. In cases of emphysema, as just observed, the emphysema disappears after a few days, the loosened bandage must be removed and replaced by a tighter one, in order to prevent displacement of the fractured ends.

Special contraindications in the use of this bandage the author sees in cases in which a binding girdle sensation is much complained of, and in advanced cases of tuberculosis of the lungs.

---

*An Objective Murmur in the Ear the Cause of Melancholy. E. Tuczek, Wochenschrift, Berlin, No.* 30, 1881.—In a lady 29 years old, who three months previously had aborted, losing much blood, there developed a state of melancholy combined with great anxiety and an inability to think. The patient grew extremely quiet, wept often, imagined her relatives were suffering want, and at last it became necessary to compel her to perform all natural duties, as eating, etc. Her sleep was interrupted by frightful dreams, and thereby a continuous roar in the ears, which patient believes to be the sole cause of all her feelings. This noise can be heard by any one in 20 ctm. of her ear, and resembles a fillip with the finger nails, or the discharge of an electric spark, This sound can be equally well heard in both ears, and occurs in a regular rhythm, and doubly as often as the heart (144 beats to 72 radial pulsations). The patient compares this sound to the ticking of a watch. Hearing good. On investigation nothing abnormal is found in or near the ear. By pressing against the posterior wall with the speculum it was discovered that the noise disappeared, and for this reason a tight-fitting tampon was introduced into the ear and allowed to remain for twenty-four hours. Immediately after the tampon was inserted the noise disappeared, and stayed away afterward, From that day the patient began to improve in feelings and appearance. After remaining four weeks in the infirmary (asylum at Marburg), she was discharged.

---

*Affections of the Joints in Tabes Dorsalis. C. Westphal. Wochenschrift, Berlin*, 1881. *No.* 38 and 39.—In connection with the report of case of deformity in both knee-joints, arising during the course of tabes, Westphal describes at length joint affections generally in tabes dorsalis. The appearance of joint affections does not seem bound to any particular period or stage of the tabes; for the most part, however, this generally appears in the later stages, but is sometimes a prodromal symptom, as in a case described in No. 37 of the *Centralblatt* for 1881. The affection of the joint runs a painless course; the skin over the joint is not even red, though sometimes this can be the case. Rarely do we find the affection in the hand, foot, toes or fingers; for the most part it is knee, hips, elbow and shoulder joint that are attacked. Westphal could not corroborate the statement made by Buzzard, that the so-called crises gastriques precede affections of the joints, for this affection of the stomach is observed at any time. The abnormal tendency to fracture, often observed and noticed, Westphal attributes to this affection in the joints, and reports a case illustrative of it.

---

*Chancres of the Vaginal Portion of the Uterus. Rasumorr, Vierteljahrschrift*, 1880, *p.* 517.—In Mjasnitzi's hospital in Moscow during the past four years, there have been treated 1,374 cases of chancre of various parts of the genital organs. Of this number 117 chancres were situate on the vaginal portion of the uterus; 13 were indurated. The anterior lip was equally affected with the posterior, however the orificium externum uteri was somewhat more frequently attacked.

In consequence of the ever accompanying cervical catarrh, the process of healing was a protracted one. The form and size of the chancres was variable, though always smaller than the chancres of the skin.

The author summarizes his experience thus: Chancres of the vaginal portion are easier to recognize, and occur more frequently than authors state, for in the majority of cases the induration was unmistakable, being cartilaginous to the touch, thus resembling the initial sclerosis. Inoculation is of no important diagnostic value.

---

*A Case of Miliary Tuberculosis Simulating Typhoid Fever. Senator, Wochenschrift,* 1881. *No.* 25.—Patient, man 42 years old, of healthy parentage, had passed through an attack of typhoid fever three years previously, and now was again attacked with a trouble showing similar symptoms, bleeding at the nose, dicrotic pulse, singultus, rose-colored spots, and in addition, enlargement of the spleen.

At last a suppurative parotitis of the left side developed. Conspicuous was only the course of the fever toward the last. At the beginning the morning remissions in the fever were greater than we are wont to observe, sometimes the difference between morning and evening being so much as 1.3°–1.2. The long duration aroused also suspicions of miliary tuberculosis, still the fundus of the eye was intact, and free of tubercles. At the obduction tubercles were everywhere to be seen, and no pathological traces of abdominal typhus.

The case is somewhat unique, having presented so many of the peculiarities and symptoms of typhoid fever.

---

*The Renal Form of Typhoid Fever. L. Homburger, Wochenschrift, Berlin,* 1881, *No.* 20.—Referring to the observations of old authors and three cases in actual experience, Homburger sets forth the following points: (1.) The diagnosis of typhoid fever can be rendered difficult through the prominence of nephritic symptoms. This is for the most part the case when these symptoms occur during the first of the attack. (2.) Gubler and Rohn have distinguished cases with prominent nephritic symptoms as the renal form. Certain it is that the physician in such cases of predominating nephritic symptoms is often puzzled to know what diagnosis to make or what treatment to establish unless he is familiar with this complex of symptoms.

Amat's theory, that the chief part of the typhus poison lay in the kidneys, has not been proved. (4.) Often the renal form resembles that observed in febrile, infectious diseases, which comes and disappears without our knowledge of its material substratum. (5) The expressed picture of nephritis, as certain other infectious diseases furnish, oftenest scarlatina, is rarely observed in the course of typhoid fever.

---

*The Effect of Work on the Secretion of Sugar and Urea in Diabetes Mellitus. H. Oppenheim, Centralblatt, No.* 1, 1881.—The experiments were made on a patient suffering with diabetes, who secreted about grm. 200=℥6½ daily. The work consisted in pumping water, and did not produce dyspnœa; between days of work were interposed several rest days. In respect to the above question there was always an indubitable increase on the work days in the amount of urea. In the first period of rest it amounted to 50 grm; on the work day 59.4 grm =℥ii ʒii. Any influence on the secretion of sugar could not be detected. In addition the author notes that the secretion of water is greater than the amount received, and that more nitrogen is secreted than is taken in with the food.

